# The effect of yttrium addition on the ratcheting behavior of magnesium

**DOI:** 10.1371/journal.pone.0348195

**Published:** 2026-06-05

**Authors:** Haoge Shou, Yaoyao Song, Yang Yang, Li Li, Mingjie Wang, Peng Shi, Qi Li, Huanjian Xie, Hongzhou Zhang

**Affiliations:** 1 Zhumadian Central Hospital affiliated of Huanghuai University, Huanghuai University, Zhumadian, China‌‌; 2 College of Intelligent Manufacturing, Huanghuai University, Zhumadian, China; 3 Logistics Service Center, Huanghuai University, Zhumadian, China; 4 Henan Topfond Pharmaceutical Co., Ltd, Zhumadian, China‌‌; University of Vigo, SPAIN

## Abstract

This study provides an in-depth investigation into the influence of yttrium (Y) addition on the ratcheting behavior of magnesium (Mg) alloys under asymmetric cyclic loading. Although pure Mg exhibits higher strength under quasi-static tension, the Mg–Y alloy demonstrates markedly superior resistance to ratcheting during cyclic deformation. The Mg–Y alloy shows a substantially reduced ratcheting strain accumulation rate accompanied by pronounced cyclic hardening, ultimately leading to a significant improvement in fatigue life. Transmission electron microscopy (TEM) reveals that the extensive activation of ⟨c + a⟩ dislocations and the resulting high dislocation density in the Mg–Y alloy play a pivotal role in promoting homogeneous plastic deformation and cyclic hardening, thereby effectively suppressing the accumulation of ratcheting strain. This work provides new insights for the development of high-performance, fatigue resistant Mg alloys.

## Introduction

Mg alloys, as the lightest structural metallic materials, hold substantial promise for applications in aerospace, transportation, and consumer electronics [1,2]. One of the critical challenges that limits their deployment in lightweight structures is their inherently poor fatigue resistance, particularly under asymmetric cyclic loading conditions where the mean stress is nonzero [3,4]. Under such loading, materials exhibit a pronounced ratcheting effect, namely, the progressive accumulation of irreversible plastic strain during each stress cycle [5]. This continual strain accumulation markedly accelerates damage evolution and can trigger premature failure at stress amplitudes far below the symmetric fatigue limit [6]. For Mg alloys with a hexagonal close-packed (HCP) crystal structure, the limited number of independent slip systems available at room temperature renders them especially susceptible to ratcheting deformation [7].

In recent years, researchers have made significant progress in improving the ratcheting and cyclic deformation properties of Mg alloys through alloying [8,9], precipitation hardening [6], thermomechanical treatments [10,11], and texture control [4]. Among these approaches, rare‑earth (RE) alloying stands out because of its unique ability to effectively weaken the basal texture and activate additional slip systems during deformation. These modifications play a crucial role not only in improving the ductility under monotonic loading but also in enhancing the resistance to cyclic damage and fatigue failure. Alloying with rare-earth (RE) elements such as yttrium (Y) can effectively weaken the basal texture and has been shown to enhance quasi-static ductility [12]. However, despite these advances, the specific influence of such texture modification on ratcheting behavior, as well as the underlying microstructural mechanisms, remains insufficiently explored. The present study aims to elucidate the effects of Y alloying on the ratcheting response of Mg alloys through systematic mechanical testing and comprehensive microstructural characterization, thereby uncovering the mechanistic origins of their ratcheting resistance, i.e., resistance to the strain accumulation rate.

## Experimental procedure

The materials investigated in this study were high-purity Mg (99.99 wt.%) and an Mg–2 wt.% Y alloy. The alloy was melted in a resistance furnace at approximately 750 °C under a protective atmosphere of SF₆ and CO₂, followed by casting into ingots. The as-cast ingots were subsequently homogenized at 400 °C for 12 h. Plate specimens were then fabricated through multi-pass hot rolling at 400 °C, reducing the thickness from 20 mm to a final value of 5 mm. All samples were sectioned from the rolled plates and subjected to a recrystallization anneal at 400 °C for 1 h to obtain fully recrystallized equiaxed microstructures. The initial microstructures, including grain size and crystallographic texture, were characterized using a Zeiss Sigma 300 field-emission scanning electron microscope (SEM) equipped with an HKL-EBSD system. The EBSD analysis was carried out with a step size of 1 μm. The scanned regions were selected from the gauge section of the specimens to ensure that the characterized microstructures are representative of the deformation region. Mechanical properties were evaluated on an Instron 8872 servo-hydraulic fatigue testing system. Quasi-static tensile tests were performed at room temperature with a strain rate of 1 × 10 ⁻ ³ s ⁻ ¹. Cyclic deformation tests were conducted in stress-control mode with a stress ratio of R = σ_min_/σ_max_ = −0.2 and −0.25, triangular waveform loading, and a frequency of 5 Hz. The gauge dimensions of both tensile and fatigue samples were 8 mm (length) × 4 mm (width) × 5 mm (thickness), as shown in S1 Fig, with the loading direction parallel to the rolling direction (RD). For each material (pure Mg and Mg–Y alloy), at least three independent specimens were tested in both the quasi-static tensile and cyclic deformation experiments to ensure the reliability and reproducibility of the results. Ratcheting strain and strain amplitude evolution were monitored by continuously recording the stress–strain hysteresis loops. The deformation-induced dislocation structures after cyclic loading were examined using a JEM-2100F TEM, operating at 200 kV. TEM foils were prepared by mechanically grinding the samples to a thickness of ~50 μm, followed by ion milling. For TEM characterization, thin foils were extracted from the gauge section, and observations were conducted on the RD–ND plane to analyze the deformation-induced microstructural features.

## Results and discussion

**Fig 1:**
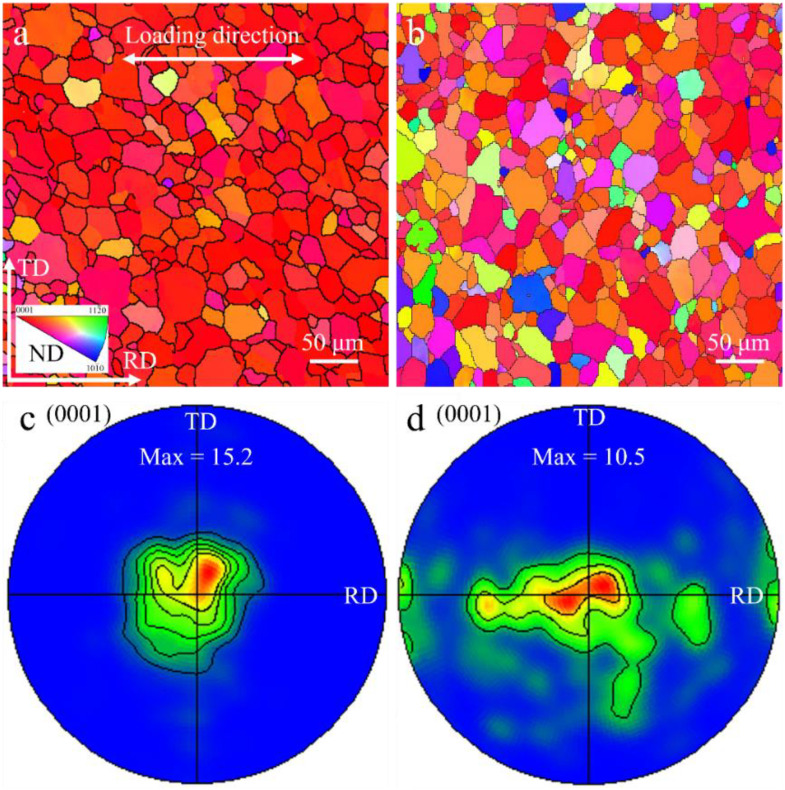
The EBSD results for the pure Mg and Mg-Y samples: (a, c) pure Mg, and (b, d) Mg-Y alloy.

Fig 1 presents the EBSD orientation maps of annealed pure Mg and the Mg–Y alloy. Both materials exhibit comparable average grain sizes of approximately ~30 μm, as shown in Fig 1a and b. However, their crystallographic textures differ markedly. Pure Mg displays a pronounced basal texture (Fig 1c), in which most (0001) planes aligned parallel to the rolling plane (RD–TD plane), manifested by the concentrated red grains in the map (Fig 1a). In contrast, the Mg–Y alloy exhibits a typical RE texture, characterized by a more dispersed grain-orientation distribution and a noticeable tilt of the ***c***-axes toward the RD direction (Fig 1d), consistent with previous reports [13]. This texture softening represents a key microstructural modification introduced by the Y element. The basal poles in pure Mg are highly concentrated along the normal direction, whereas in the Mg–Y alloy, they exhibit a pronounced spreading toward the RD direction. This redistribution of texture components provides favorable conditions for activating additional slip systems under cyclic loading.

**Fig 2:**
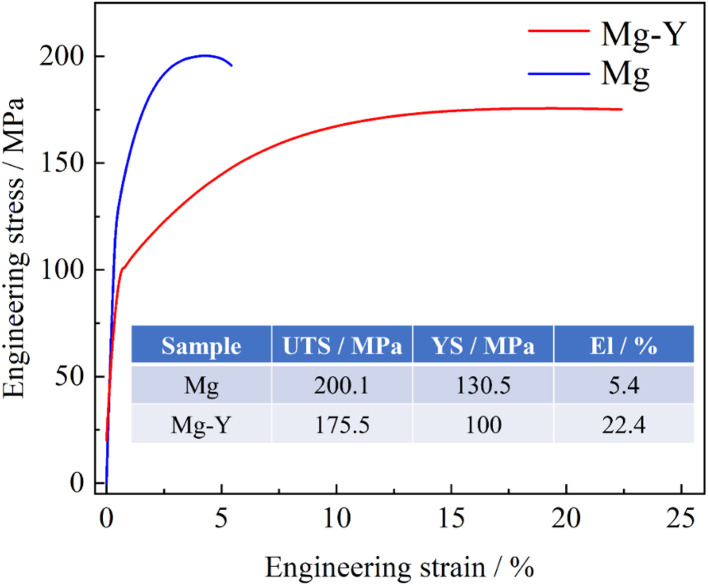
Tensile engineering stress-strain curves of the pure Mg and Mg-Y alloys.

The engineering stress–strain curves obtained under quasi-static tension (Fig 2) clearly reveal the influence of texture on the mechanical response. As summarized in the table, pure Mg exhibits a yield strength (YS) of 130.5 MPa and an ultimate tensile strength (UTS) of 200.1 MPa, but a limited elongation (EI) of only ~5.4%. In contrast, the Mg–Y alloy shows reduced YS and UTS values of 100 MPa and 175.5 MPa, respectively, whereas its EI markedly increases to 22.4%. The high strength but poor ductility of pure Mg originates from its strong basal texture [14]. During tension along the RD, the Schmid factor for basal slip is extremely low, making deformation difficult and leading to elevated YS but insufficient plastic reserve. In comparison, the RE texture formed in the Mg–Y alloy places a larger fraction of slip systems (particularly basal slip) in favorable orientations during RD loading. This results in pronounced “texture softening” at yielding, lowering the YS while significantly enhancing the uniformity of plastic deformation and thus improving ductility, consistent with the findings reported by Li et al. [13].

**Fig 3:**
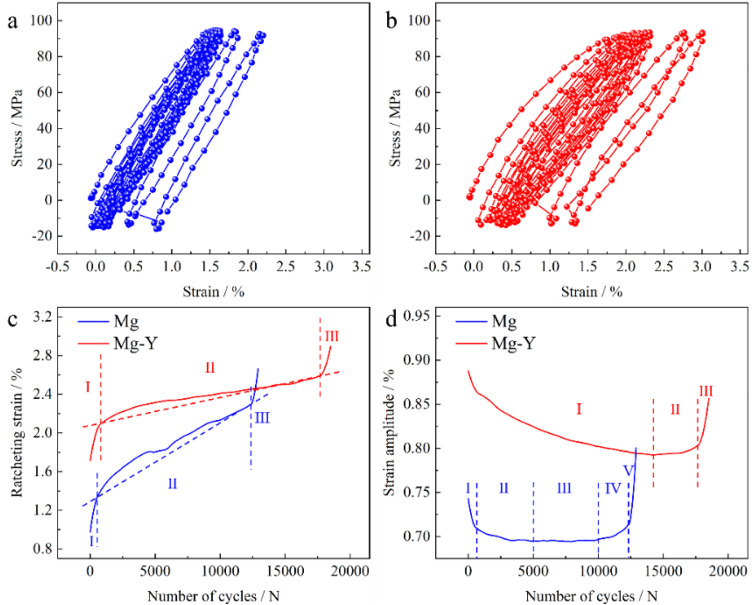
Stress-strain response for the (a) pure Mg at σ_max_ = 100 MPa, R = −0.2, and (b) Mg-Y alloy at σ_max_ = 100 MPa, R = −0.2, the evolution of (c) ratcheting strain, and (d) strain amplitude with the cycle numbers.

This difference in monotonic deformation characteristics directly translates to the cyclic loading response. To clarify the influence of Y on ratcheting behavior, the cyclic stress–strain responses of both samples were monitored in detail. Fig 3 presents the deformation responses of the two samples under cyclic stress-controlled loading. The stress–strain hysteresis loops in Fig 3a and b clearly demonstrate that the Mg–Y alloy exhibits more saturated and fuller loop shapes, indicating a larger amount of plastic strain energy absorbed per cycle, which is consistent with its superior ductility under quasi-static loading. Meanwhile, the broader area enclosed by the hysteresis loops in the Mg–Y alloy also signifies enhanced energy dissipation capability during cyclic deformation, delaying fatigue damage evolution and crack propagation. In addition, under identical loading conditions, the Mg–Y sample (18,505 cycles) shows a markedly longer fatigue life compared with pure Mg (13,029 cycles).

The evolution of ratcheting strain (ε_r_ = (ε_max_ + ε_min_)/2) with the number of cycles (N), shown in Fig 3c, reveals the most critical distinction between the two materials. Ratcheting strain is defined as the residual tensile plastic strain accumulated at the end of each cycle during the tensile half-cycle [3,5]. Pure Mg exhibits a rapid accumulation of ε_r_ from the initial stage (stage I), and enters the steady ratcheting stage (stage II) within only ~200 cycles, with almost no apparent saturation stage. This accelerated ratcheting process demonstrates that pure Mg lacks the capacity to continuously harden under cyclic loading, leading to premature failure. The material reaches failure rapidly, exhibiting a high ratcheting strain rate (dε_r_/dN) and a poor resistance to ratcheting deformation. Such accelerated strain accumulation is the primary reason for its shortened fatigue life. In sharp contrast, the accumulation of ratcheting strain in the Mg–Y alloy is effectively suppressed. Its ratcheting strain rate is significantly lower than that of pure Mg, and the steady ratcheting stage (stage II) is substantially prolonged, highlighting its excellent resistance to ratcheting deformation. The delayed transition to the ratcheting saturation stage and the lower strain slope reflects its enhanced cyclic stability and ratcheting resistance. These features indicate that the addition of Y effectively reduces the rate of ratcheting strain accumulation.

Further evidence is provided by Fig 3d, which shows the evolution of strain amplitude (Δε/2). For pure Mg, Δε/2 decreases slightly during the early cycles but then remains nearly constant or increases slowly, suggesting that its strain hardening capability is rapidly exhausted and becomes insufficient to counteract the continued accumulation of plastic strain [2,15]. This trend directly corresponds to the rapid increase in ratcheting strain. In contrast, the Mg–Y alloy exhibits a pronounced and continuous decrease in Δε/2 throughout almost the entire cyclic process (stages I and II), a characteristic signature of cyclic hardening. Such cyclic hardening elevates the flow stress progressively during cyclic loading, requiring a higher driving force to generate the same amount of plastic strain [16]. Consequently, it effectively suppresses the accumulation of ratcheting strain and accounts for the significantly enhanced ratcheting resistance and prolonged fatigue life in the Mg–Y alloy. To further evaluate the robustness of the ratcheting behavior, additional cyclic tests were conducted under different loading conditions. As shown in S2 Fig, at a higher stress level (σ_max_ = 140 MPa, R = −0.2), the fatigue life increases from 6840 cycles for pure Mg to 8280 cycles for the Mg–Y alloy, corresponding to an improvement of approximately 21%. Under the lower stress condition (σ_max_ = 80 MPa, R = −0.25), the fatigue life is extended from 25,200 cycles to 39,600 cycles, representing an improvement of approximately 57%. In both cases, ratcheting strain accumulation is observed in both Mg and Mg–Y, however, the Mg–Y alloy exhibits stronger resistance to ratcheting and a longer fatigue life.

**Fig 4:**
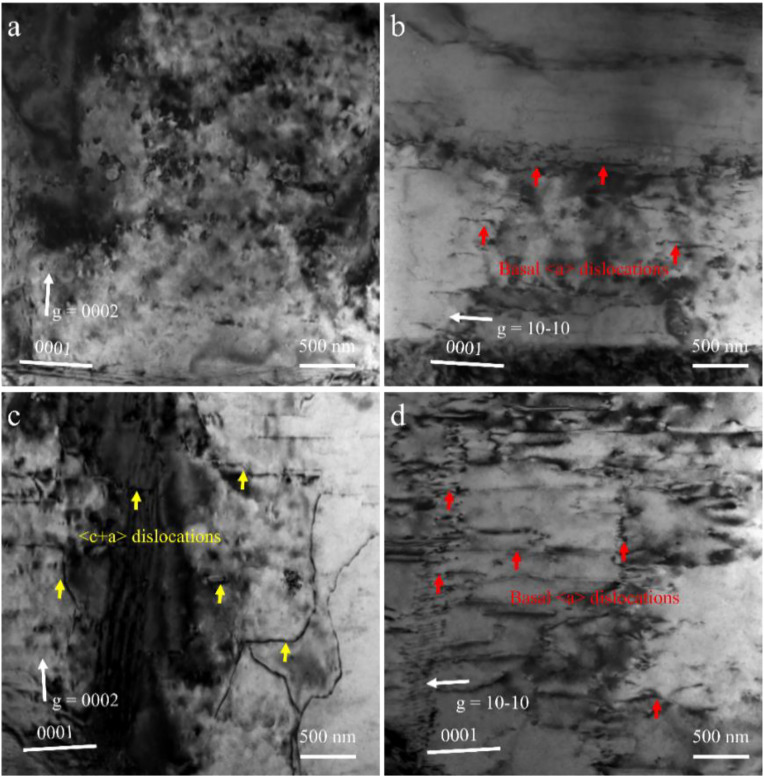
Typical dislocation configuration after fatigue in the pure Mg and Mg-Y alloys: (a) and (b) TEM images viewed with diffraction vectors were *g* = 0002 and *g* = 10−10 of the pure Mg, respectively, (c) and (d) TEM images viewed with diffraction vectors were *g* = 0002 and *g* = 10−10 in the Mg-Y alloy, respectively.

The tensile curves clearly show that the Mg–Y alloy exhibits a distinct yield plateau, which is attributed to the activation of a limited amount of {10–12} extension twinning at the early stage of deformation. EBSD analysis at a strain of ~0.5% (S3 Fig) confirms the presence of a small fraction of extension twins, indicating their contribution to the initial plastic strain accommodation. The activation of extension twinning can be primarily attributed to the texture, where a fraction of grains has their ***c***-axes oriented nearly parallel to the RD (Fig 1d). With increasing strain, deformation gradually transitions to dislocation-dominated plasticity, particularly involving non-basal ⟨c + a⟩ slip systems, leading to sustained work hardening. This sustained strain-hardening capability effectively facilitates cyclic strain accommodation, promotes cyclic hardening, and suppresses localized strain accumulation. To further elucidate the microstructural mechanisms, TEM observations were performed. Fig 4 presents typical dislocation configurations after cyclic loading. For pure Mg with a pronounced basal texture (Fig 1a and c), the number of activated slip systems during cyclic loading along the RD is severely limited, leading to highly localized deformation [17]. Such deformation heterogeneity results in rapid dislocation accumulation and damage formation within confined regions, while offering little contribution to macroscopic hardening. These localized dislocation structures are more likely to generate stress concentrations near grain boundaries or weak interfaces, accelerating crack initiation and propagation. Consequently, ratcheting strain grows in an uncontrolled manner. In contrast, the Mg–Y alloy exhibiting a RE-type texture shows a substantial tilt of the grain ***c***-axes toward the RD. Under cyclic loading along the RD, this texture modification not only facilitates the activation of basal <a> slip, but more importantly, enables widespread activation of non-basal slip modes (particularly <c + a> slip). This behavior is primarily attributed to the reduced CRSS_non-basal_/ CRSS_basal_ ratio induced by Y addition [18,19]. After cyclic loading, the microstructure of pure Mg is dominated by basal <a> dislocations (Fig 4a and b). However, in the Mg–Y alloy, besides the high density of basal <a> dislocations, abundant <c + a> dislocations are clearly observed (Fig 4c, highlighted by yellow arrows). The presence of <c + a> dislocations is critical, as they accommodate strain along the ***c***-axis and thereby promote deformation compatibility. The activation of a larger number of slip systems (particularly <c + a> slip) offers two key advantages: (1) it promotes more homogeneous plastic deformation, mitigates local stress concentrations, and delays fatigue crack initiation; (2) the complex interactions among various dislocation types (e.g., junction formation and entanglement) generate pronounced cyclic hardening (Fig 3d), which effectively suppresses the accumulation of ratcheting strain. Furthermore, the dislocation networks act as barriers to subsequent dislocation motion, thereby enhancing the cyclic stability and delaying ratcheting damage. As a result, despite its lower YS under different cyclic loading conditions, the Mg–Y alloy exhibits superior resistance to ratcheting deformation and a significantly prolonged fatigue life. Through texture modification and the promotion of multi-slip activation (especially <c + a> slip), the addition of Y fundamentally optimizes the deformation mechanisms of Mg alloys under cyclic loading.

## Conclusions

This study provides a systematic comparison of the microstructures, quasi-static mechanical responses, and ratcheting cyclic deformation behaviors of pure Mg and the Mg–Y alloy, and elucidates the underlying deformation mechanisms through TEM characterization. The major conclusions are summarized as follows:

(1) The addition of Y markedly modifies the texture of Mg alloys, transforming the strong basal texture of pure Mg into a RE-type texture in the Mg–Y alloy, where the ***c***-axes tilt toward the RD. This texture transition reduces the YS but significantly enhances ductility.
(2) Under asymmetric cyclic loading along the RD, the Mg–Y alloy exhibits a markedly superior resistance to ratcheting deformation compared with pure Mg. Pure Mg shows a high ratcheting strain rate, whereas the Mg–Y alloy demonstrates a substantially lower ratcheting strain rate accompanied by pronounced cyclic hardening.
(3) During cyclic loading, pure Mg primarily accommodates deformation through limited basal <a> slip, which readily leads to stress concentration. In contrast, Y addition promotes the activation of multiple slip systems. TEM observations reveal a high density of dislocations, including abundant <c + a> dislocations in the Mg–Y alloy. These dislocation structures promote uniform plastic deformation, increase cyclic hardening, and suppress ratcheting strain accumulation. The activation of <c + a> dislocations is critical for achieving homogeneous plastic deformation and cyclic hardening, it enhances strain compatibility, retards damage accumulation, and ultimately improves the ratcheting resistance and lifetime of the Mg–Y alloy.

Overall, this study demonstrates that the Y element, through texture modification and the promotion of <c + a> slip activation, constitutes an effective strategy for enhancing the fatigue performance of Mg alloys. These findings provide important mechanistic insights and experimental evidence for the development of high fatigue resistant Mg alloys.

## Supporting information

S1 Fig Drawings of the dog-bone samples used for fatigue and tensile tests with dimensions given in millimeters. Note: Tensile and fatigue tests were performed on dog-bone samples machined according to ASTM.
(DOCX)

S2 Fig Stress-strain response for the (a) pure Mg at σ_max_ = 80 MPa, R = −0.25, and (b) Mg-Y alloy at σ_max_ = 80 MPa, R = −0.25, the evolution of (c) ratcheting strain. (DOCX)

S3 Fig EBSD results of the Mg–Y alloy at a tensile strain of 0.5%. (DOCX)
