# Supplementary material for: The effect of yttrium addition on the ratcheting behavior of magnesium
Source: PLoS One. 2026 Jun 5;21(6):e0348195. doi: 10.1371/journal.pone.0348195 (PMC13240880; doi:10.1371/journal.pone.0348195)
Supplement: S1 Fig — Note: Tensile and fatigue tests were performed on dog-bone samples machined according to ASTM. (DOCX) [file pone.0348195.s001.docx]

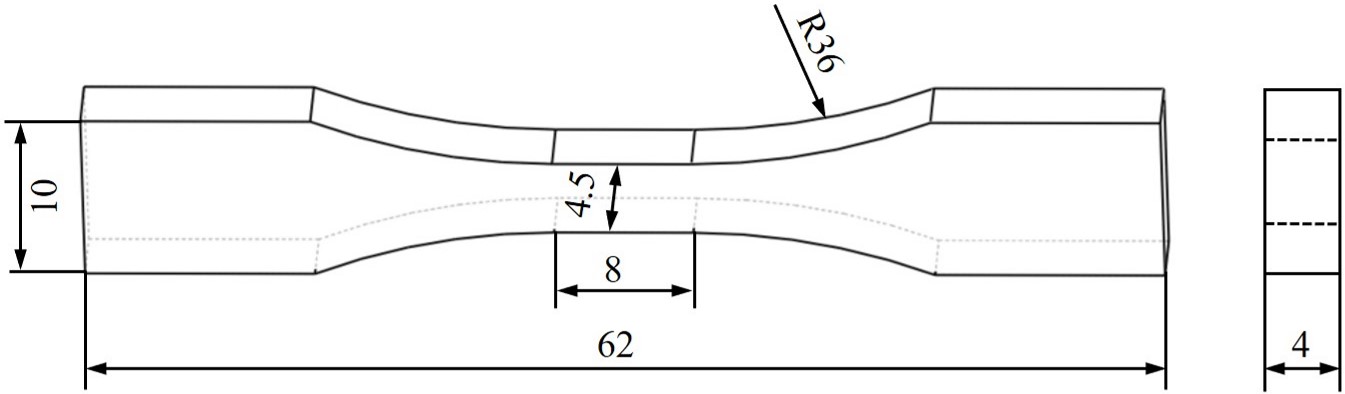


**Fig. S1.** Drawings of the dog-bone samples used for fatigue and tensile tests with dimensions given in millimeters. Note: Tensile and fatigue tests were performed on dog-bone samples machined according to ASTM.
