# Supplementary material for: The effect of yttrium addition on the ratcheting behavior of magnesium
Source: PLoS One. 2026 Jun 5;21(6):e0348195. doi: 10.1371/journal.pone.0348195 (PMC13240880; doi:10.1371/journal.pone.0348195)
Supplement: S2 Fig — (DOCX) [file pone.0348195.s002.docx]

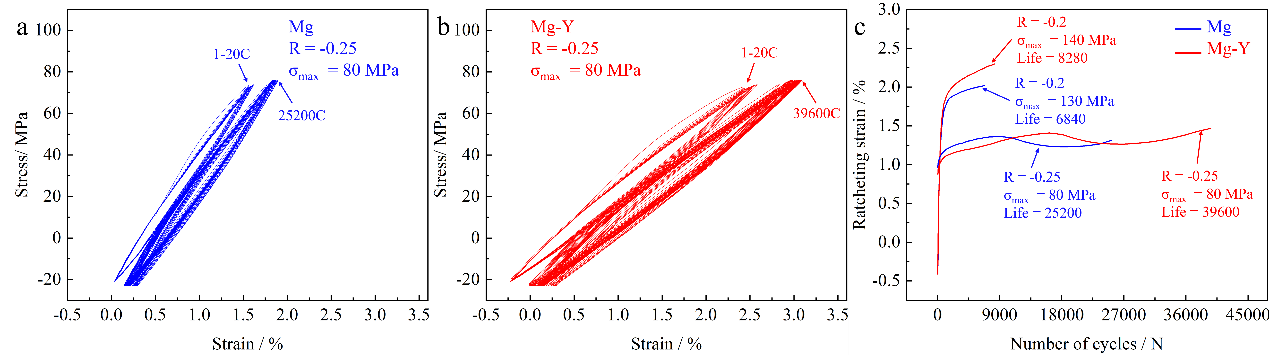


**Fig. S2.** Stress-strain response for the (a) pure Mg at σ_max_ = 80 MPa, R = −0.25, and (b) Mg-Y alloy at σ_max_ = 80 MPa, R = −0.25, the evolution of (c) ratcheting strain.
