# Supplementary figures and images for: The effect of yttrium addition on the ratcheting behavior of magnesium
Source: PLoS One. 2026 Jun 5;21(6):e0348195. doi: 10.1371/journal.pone.0348195 (PMC13240880; doi:10.1371/journal.pone.0348195)

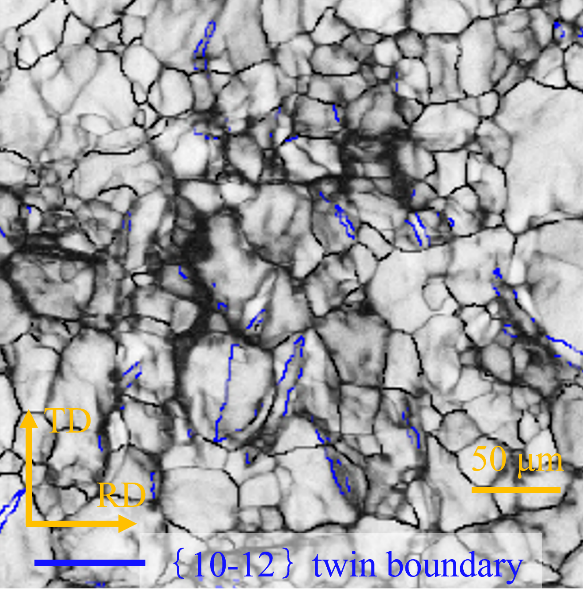


**Fig. S3.** EBSD results of the Mg–Y alloy at a tensile strain of 0.5%.

Supplement: S3 Fig — (DOCX) [file pone.0348195.s003.docx]
